# Ultrasound Elastography under Deep Learning Algorithm to Analyze the Therapeutic Effect of Clustered Regularly Interspaced Short Palindromic Repeats Short Hairpin Ribonucleic Acid Nanoparticles on Cervical Cancer

**DOI:** 10.1155/2021/7538984

**Published:** 2021-11-29

**Authors:** Minghui Li, Weiwei Li, Liang Zhao

**Affiliations:** Department of Ultrasound Diagnosis, Affiliated Tumor Hospital of Xinjiang Medical University, Urumqi 830011, Xinjiang, China

## Abstract

This study aimed to analyze the effect of the deep learning algorithm on ultrasound elastography on the treatment of cervical cancer with clustered regularly interspaced short palindromic repeats (CRISPR) short hairpin ribonucleic acid (shRNA) nanoparticles, aiming to provide a reference for the clinical application of deep learning to analyze the therapeutic effect of the disease. In this study, CRISPR and shRNA plasmid nanoparticle drugs were used to treat 55 patients with cervical cancer in the experimental group, and normal saline was injected to another 53 patients in the control group, so compare the effect of nanoparticles in the treatment of cervical cancer. Professional doctors and the recurrent neural network (RNN) intelligent algorithm were used to score cervical cancer based on the ultrasound elastograph images by taking blue, green, and red (BGR) as diagnosis criteria. As a result, the experimental group had a total of 217 points before drug administration and a total of 224 points after drug administration. Each patient had an average increase of 0.13 points. The control group had a total of 200 points before drug administration and a total of 223 points after drug administration, and each patient had an average increase of 0.43 points. The experimental group was obviously different from the control group (*P* < 0.05). Each tissue image output by the RNN was clearer than the original image, and the score given by intelligent calculation was faster than that of professional doctors. The monitoring effect of the deep learning RNN intelligent algorithm on the therapeutic effect of nanomedicine was analyzed. It was found that the average accuracy of the experimental group and the control group was 98.95% and 90.34%, respectively; and the experimental group was greatly different from the control group (*P* < 0.05). In short, nano-CRISPR and shRNA drugs had remarkable effects on the treatment of cervical cancer, and the scores given by the deep learning intelligent algorithm were faster and more accurate, which provided theoretical guidance for the clinical application of deep learning algorithms to analyze the treatment effects of diseases.

## 1. Introduction

Cervical cancer is a common gynecological malignant tumor [1]. Carcinoma in situ is concentrated in the age of 30–35, and invasive cancer is concentrated in the age of 45–55. There is a trend of younger generation. With the development of imaging, examinations are becoming more and more common, enabling early detection and early treatment of cervical cancer, and its mortality significantly decline [2]. The etiology is related to viral infection, sexual behavior, number of childbirths, and other factors. Cervical smear cytology is the main method of cervical cancer screening. Others include the cervical iodine test and colposcopy. However, in some economically underdeveloped areas, early screening for cervical cancer is not universal, and women who have been vaccinated against human papillomavirus (HPV) are still at risk of getting the disease. Therefore, it is necessary to study a new type of drug to treat HPV to achieve the purpose of reversing cervical tissue lesions.

In recent years, with the development of the material field, nanomedicine has been widely reported in the treatment of cervical cancer. CRISPR is a repetitive sequence in the genome of prokaryotes, which can integrate its own genes into the genes of bacteria, and bacteria have evolved the CRISPR-Cas9 system to understand foreign genes [3]. It can make the genome more effective to produce changes or mutations, and the efficiency is higher than other gene editing techniques, but it may produce a large number of accidental targets in human cancer cells [4]. shRNA has two short inverted repeats, separated by a stem-loop sequence to form a hairpin structure [5]. When small interfering RNA is delivered in vivo, the siRNA sequence is cloned into a plasmid vector as a short hairpin; when it is delivered to an animal body, the hairpin sequence is expressed to form double-stranded RNA and processed by the RNAi channel. Transfection of cervical cancer tissue with nanoparticles containing clustered regularly interspaced short palindromic repeats (CRISPR) and short hairpin RNA (shRNA) plasmids can effectively inhibit the growth of cervical cancer cells [6].

Ultrasound-assisted elastography (ETE) can be roughly divided into two categories: intravascular elastography and tissue elastography, which can be used to analyze the therapeutic effect of cervical cancer [7]. Deep learning is a new research direction in the field of machine learning; it has achieved results in speech and image recognition, search technology, data mining, machine learning translation, and other related fields and has solved many complex pattern recognition problems and made great progress in artificial intelligence-related technologies [8]. The recurrent neural network (RNN) is one of the common deep learning algorithms. The RNN takes sequence data as input, recursively in the evolution direction of the sequence, and all nodes (cyclic units) are connected in a chain [9]. In this study, 108 patients with cervical cancer were randomly divided into an experimental group with 55 patents treated with CRISPR and shRNA plasmid nanoparticle drugs and a control group with 53 patients treated with the same amount of normal saline. The ultrasound elasticity of the two groups before and after administration was collected. In addition, the imaging results were compared to analyze the effect of CRISPR shRNA nanoparticles in the treatment of cervical cancer, aiming to provide data support and theoretical guidance for the clinical diagnosis and treatment of cervical cancer.

## 2. Materials and Methods

### 2.1. Research Objects and Their Grouping

In this study, 108 cervical cancer patients diagnosed by colposcopy biopsy in the hospital from January 2018 to January 2020 were selected as the research objects, with an average age of 43.2 ± 8.9 years old. 55 cases were randomly selected as the experimental group, and the remaining 53 cases were undertaken as controls. This study had been approved by the ethics committee of the hospital, and the patients and their families had understood the content and methods of the study and agreed to sign the corresponding informed consents.

The inclusion criteria were defined as follows: patients whose age was between 18 and 65, patients who were diagnosed with cervical cancer after clinical diagnosis, patients who had not received other drug treatment recently, patients had not received chemotherapy and radiotherapy, and patients who had no history of other treatments in the reproductive system.

The exclusion criteria were defined as follows: patients who had undergone tumor resection, patients with incomplete clinical data, and patients who did not cooperate with doctors throughout the treatment.

### 2.2. Nanoparticle Preparation and Drug Administration of CRISPR and shRNA

The poly *β*-amino ester (PBAE) and CRISPR/shRNA plasmid DNA were diluted in 25 mM of (pH = 5) sodium acetate solution. According to the different mass ratio (PBAE to plasmid), the PBAE solution was added dropwise to the same volume of the plasmid-containing solution and mixed gently for 30 seconds. The mixture was allowed to stand at room temperature for 15 minutes to completely synthesize PBAE/nanoparticles of plasmid deoxyribonucleic acid (DNA). 1% pentobarbital solution was intraperitoneally injected. The synthesized nanoparticles (containing 10 ug of plasmid) were injected into the vagina of patients with cervical cancer in the experimental group. Among the 55 cases in the experimental group, 25 cases were injected with PBAE/CRISPR plasmid DNA nanoparticle drugs, and 30 cases were injected with PBAE/shRNA plasmid DNA nanoparticle drugs, once a day for 20 days. The control group was injected with the same amount of normal saline, and then, the changes in the ultrasound elastography scores of the experimental group were observed.

### 2.3. Ultrasound Elastography

All patients underwent ultrasound elastography examination after vaginal ultrasound examination. The probe was moved to the cervix to observe the size of the cervix, the capsule, the characteristics of the gray-scale ultrasound, and the blood flow velocity of the cervix. Then, the probe was moved to the vaginal fornix and lightly attached to the external cervix for testing, the duration was 3–5 s, and the color coding ranged 0–180 kPa. The measurement should be repeated for 3 times to record and calculate the average of each group of data. All images were completed by an experienced professional imaging physician.

### 2.4. RNN under Deep Learning Algorithm

**Figure 1:**
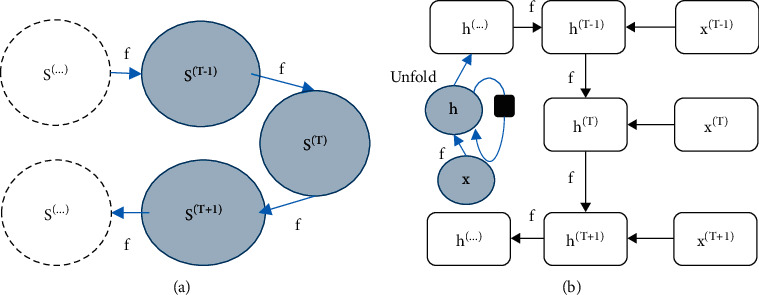
Dynamic system diagram. (a) A dynamic system without input. (b) A dynamic system with input.

Convolutional neural network (CNN) models are widely used in image classification. Among them, the network neurons in the same layer are not independent of each other, but have a certain correlation. In the RNN model, the convolutional layer is built by combining the single-loop neural network and the convolutional layer, and the sampling layer is consistent with the same output layer. A simple RNN consisted of an input layer, a hidden layer, and an output layer. Its dynamic system had an input dynamic system and an input dynamic system, as shown in Figure 1. The equations of the dynamic system without input are (1) and (2); the equations for the dynamic system with input are (3) and (4). *f* represents the unit operation, the final state of the dynamic system with input is represented as *h*, the final state of the dynamic system without input is represented as S, *X* represents the input, *T* represents the state of the unit, *T*+1 and *T*−1 represent the increase or decrease of the unit state, and the ellipsis indicates the follow-up process can continue to run as such.

(1) $$\begin{array}{c} S^{\left(T\right)}=f\left(S^{\left(T-1\right)};\theta \right),\\ S^{\left(3\right)}=f\left(S^{\left(2\right)};\theta \right)=f\left(f\left(S^{\left(1\right)};\theta \right);\theta \right),\\ h^{\left(T\right)}=f\left(h^{\left(T-1\right)},X^{\left(T\right)};\theta \right),\\ h^{\left(T\right)}=g^{\left(T\right)}\left(X^{\left(T\right)},X^{\left(T-1\right)},X^{\left(T-2\right)},\ldots ,X^{\left(2\right)},X^{\left(1\right)}\right)=f\left(h^{\left(T-1\right)},X^{\left(T\right)};\theta \right). \end{array}$$

**Figure 2:**
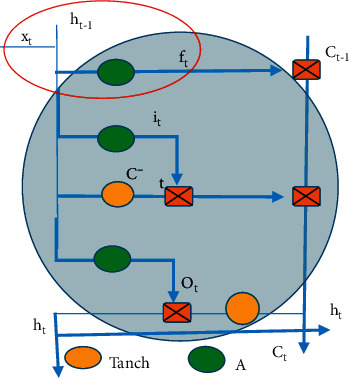
Diagram for location of the forgotten gate.

The following RNN algorithm was proposed by Hinton in 2015 [10], which uses ReLU to activate neurons and initializes the weights of the entire network to make the final model effect better than most existing algorithms. The location of the forget gate in the algorithm flowchart is shown in Figure 2, and the red circle area is shown in equation (2). *f* represents the internal unit operation, *t* represents the state of the unit, the unit operation on the state is defined as function *f*_*t*_, A and Tanc represent the inputs of the vector, the final state of the forget gate is represented as *h*, the final state of the input gate is expressed as *i*, the final state of the total input is represented as C, O refers to the final state of the output gate, and T-1 represents the reduction of the unit state.

(2) $$\begin{array}{c} f_{t}=A\left(W_{fx}x_{t}+W_{Th}h_{t-1}+b_{f}\right). \end{array}$$

**Figure 3:**
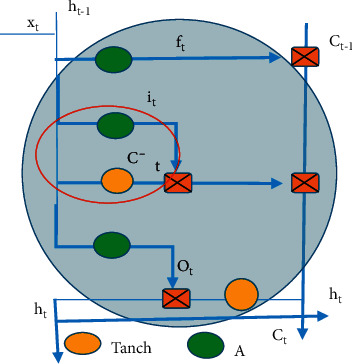
Diagram for location of the input gate.

The input gate positioning in the algorithm flowchart is shown in Figure 3 (the red circle area), and the equations are given as follows:

(3) $$\begin{array}{c} C_{t}^{\prime }=Tanh\left(W_{cx}x_{t}+W_{ch}h_{t-1}+b_{c}\right),\\ i_{t}=A\left(W_{ix}x_{t}+W_{Th}h_{t-1}+b_{i}\right). \end{array}$$

**Figure 4:**
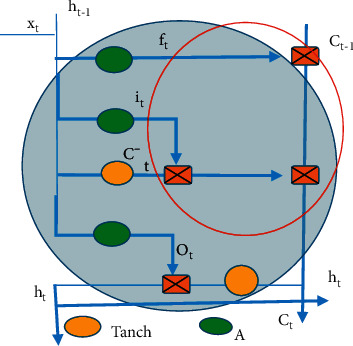
Diagram for location of the total input.

The total input positioning in the algorithm flowchart is shown in Figure 4 (the red circle area) and equation (4).

(4) $$\begin{array}{c} C_{t}=f_{t}*C_{t-1}+i_{t}*C^{\prime }. \end{array}$$

**Figure 5:**
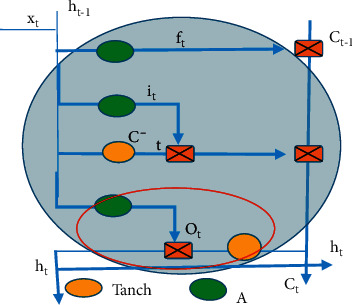
The location of the output gate in the algorithm flowchart.

The location of the output gate in the algorithm flowchart is shown in Figure 5 (the red circle area) and equations (5) and (6).

(5) $$\begin{array}{c} o_{t}=A\left(W_{ox}x_{t}+W_{oh}h_{t-1}+b_{o}\right), \end{array}$$

(6) $$\begin{array}{c} h_{t}=o_{t}*\text{Tanh}\left(C_{t}\right)\left(f\right). \end{array}$$

### 2.5. The Treatment and Efficacy Evaluation

ETE image evaluation was given as follows. The elasticity diagram used different colors to represent the hardness of different tissues, in which red indicates the average hardness was softer and the tissue was softer, green indicates the average elasticity of the tissue, and blue indicates the average hardness was harder. Generally, in normal cervical imaging, the display is mostly blue and green, and a small part of it is red. The specific scoring method studied in this study was defined as follows. If blue, green, and red were visible and blue area was ≤50%, the score was 1 point; if blue and green alternated without red and the blue area was ≤50%, the score was 2 points; if blue and green were visible with no red and the blue area was 50%–70%, the score was 3 points; if the image display was mainly blue, with a small part of green and no red and the blue area was more than 70%, the score was 4 points; and if the entire area of the cervix was shown in uniform blue, the score was 5 points.

In order to quantitatively evaluate the performance of the algorithm in this study, three commonly used evaluation indicators in medical image segmentation were used as the standard to measure the results of this experiment. Accuracy represented the ratio of all correctly predicted pixels to the total number of pixels. The specific calculation method is shown in equation (7), where TP represents the number of true positive, FP represents the number of false positive, FN represents the number of false negative, and TN represents the number of true negative.

(7) $$\begin{array}{c} A=\frac{\text{TP}+\text{TN}}{\text{TN}+\text{TP}+\text{FP}+\text{FN}}. \end{array}$$

### 2.6. Statistical Methods

The software used for data processing in this study was SPSS 19.0. For statistical analysis, measurement data were expressed as mean ± standard deviation ($\bar{x}$ ± *s*), and count data were expressed as percentage (%). Pairwise comparison used analysis of variance. *P* < 0.05 means the difference was statistically significant.

## 3. Results

### 3.1. The Therapeutic Effect of CRISPR and shRNA Plasmid Nanoparticles on Cervical Cancer

**Figure 6:**
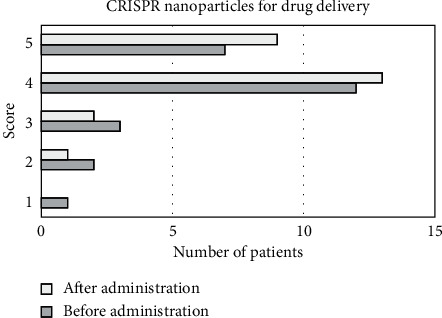
The CRISPR nanoparticles drug administration score for patients in the experimental group.

As shown in Figure 6, 25 image scores of CRISPR nanoparticles before the drug administration were randomly selected, including 1 patient with 1 point, 2 patients with 2 points, 3 patients with 3 points, 12 patients with 4 points, and 7 patients with 5 points. After drug administration, there were 0 patient at 1 point, 1 patient at 2 points, 2 patients at 3 points, 13 patients at 4 points, and 9 patients at 5 points.

**Figure 7:**
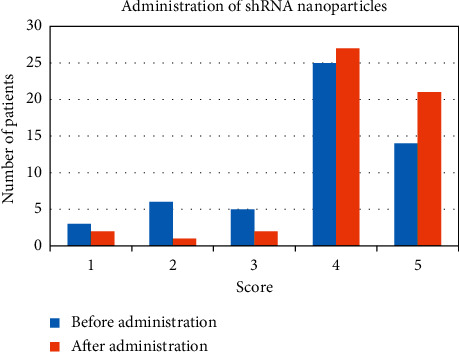
The drug administration score of shRNA nanoparticle of the experimental group.

As shown in Figure 7, 30 cases of imaging scores before shRNA nanoparticle drug administration were randomly selected from the experimental group; there were 1 patient with 1 point, 3 patients with 2 points, 4 patients with 3 points, 14 patients with 4 points, and 8 patients with 5 points; after drug administration, there was 1 patient at 1 point, 2 patients at 2 points, 3 patients at 3 points, 15 patients at 4 points, and 9 patients at 5 points.

**Figure 8:**
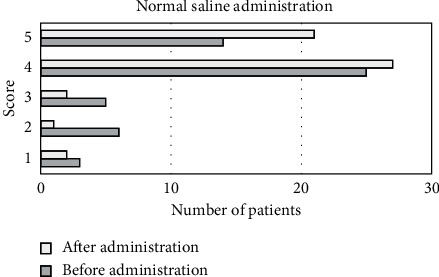
The scoring of drug administration with normal saline in the control group.

As shown in Figure 8, the image scores of the control group before drug administration were 3 patients at 1 point, 6 patients at 2 points, 5 patients at 3 points, 25 patients at 4 points, and 14 patients at 5 points. After drug administration, there were 2 patients at 1 point, 1 patient at 2 points, 2 patients at 3 points, 27 patients at 4 points, and 21 patients at 5 points.

**Figure 9:**
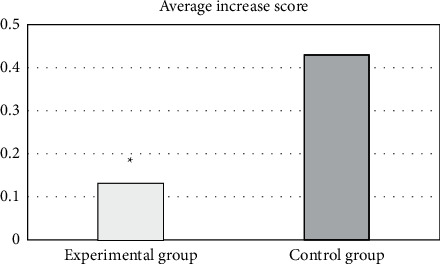
The scoring of drug administration with normal saline in the control group. ^*∗*^The difference was statistically great in contrast to the control group (*P* < 0.05).

As shown in Figure 9, the experimental group had a total of 217 points before drug administration and a total of 224 points after drug administration, with an average increase of 0.13 points per patient, and the control group had a total of 200 points before drug administration and a total of 223 points after drug administration, with each patient having an average increase of 0.43 points. Therefore, the experimental group was greatly different from the control group (*P* < 0.05).

### 3.2. Image Analysis of Ultrasound Elastography under RNN

**Figure 10:**
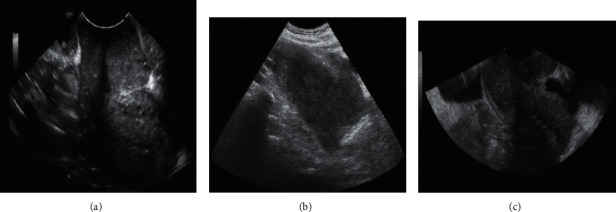
Traditional ultrasound image of cervical cancer. (a) An image of the early symptoms of cervical cancer. (b) The uterine cavity is closed and the periphery of the cervical lesion is blurred. (c) The cervical lesions are visible.

Figure 10 shows a traditional ultrasound image of three random patients. The image was not in color. It can roughly analyze cervical cancer qualitatively, but it was difficult to analyze cervical cancer quantitatively. With the development of technology, ultrasonic elastic imaging can simultaneously perform qualitative and quantitative analyses of cervical cancer.

**Figure 11:**
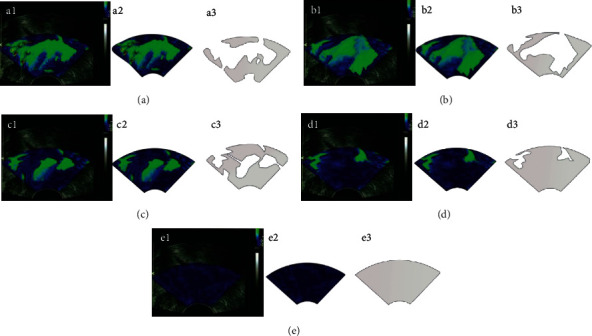
Diagram of ultrasonic elastic imaging analysis under the deep learning algorithm.

Figure 11 shows the analysis process of the algorithm's influence on ultrasound elastography. The following figure is the image of five random patients, and A, B, C, D, and E in the figure refer to five different scores of 1–5, respectively. Figures A1, B1, and C1 are the original images of ultrasonic elastic imaging, and different colors represent tissues of different hardness. Figures A2, B2, and C2 show tissue images output by the deep learning algorithm based on the original ultrasound elastography image. Compared with the original image, the hardness of the tissue can be clearly distinguished. Figures A3, B3, and C3 show the blue area of the hardness output by the deep learning algorithm. The score was given through the calculation of the intelligent algorithm. Compared with professional doctors, this method was faster and more accurate.

**Figure 12:**
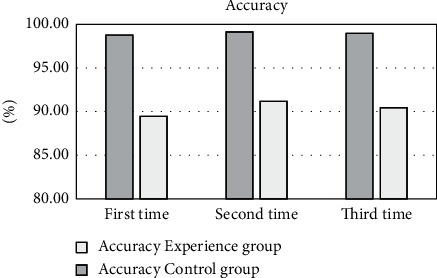
The comparison on accuracy in the experimental group and the control group.

The scores given by professional doctors and RNN intelligent algorithms were compared to analyze the monitoring effect of deep learning on the therapeutic effect of nanomedicine. Each test was performed three times, as shown in Figure 12. The data showed that the accuracy of the experimental group was 98.76%, 99.13%, and 98.96% for three times, respectively, and that for the control group was 89.43%, 91.15%, and 90.45%, respectively.

**Figure 13:**
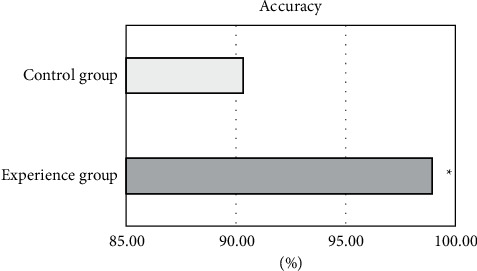
Comparison on average accuracy between the experimental group and the control group. ^*∗*^The difference was statistically great in contrast to the control group (*P* < 0.05).

As shown in Figure 13, the average accuracy of the experimental group was 98.95%; the average accuracy of the control group was 90.34% and that in the experimental group was greatly different from that in the control group (*P* < 0.05).

## 4. Discussion

Persistent high-risk HPV infection is the main risk factor for cervical cancer. Even if the HPV vaccine has been vaccinated, the risk of other high-risk HPV infections faced by women still exists. Although the HPV vaccine can stimulate the body to produce antibodies to avoid infection, its therapeutic effect is minimal for women who already have cervical disease. Cervical screening can also be done on a regular basis, but in some economically underdeveloped areas, this type of examination is not universal and easy to achieve. Therefore, considering the actual needs of patients threatened by the HPV virus, this study combined PBAE and CRISPR shRNA to design a nanoparticle drug. In the field of molecular biology today, the silencing technology represented by shRNA can bind to mRNA encoding the protein at the mRNA level, so that the gene can be specifically degraded [11]. Knockout technology represented by CRISPR uses double-stranded DNA cutting and mismatch repair to prevent specific genes from achieving normal transcription and translation at the DNA level [12, 13]. Since these functional plasmids cannot penetrate the body's organs and tissues by themselves, an appropriate carrier system is needed [14]. In this study, PBAE with low toxicity, good degradability, high transfection efficiency, and excellent stability was used to complete plasmid delivery. It has currently shown good efficacy in the clinical treatment of some cancers.

Ultrasound-assisted elastography technology can detect tumors and spreading diseases that cannot be detected by traditional ultrasound and is an important imaging method to analyze and detect the therapeutic effect of cervical cancer [15, 16]. The RNN is one of the common deep learning algorithms. It is a type of algorithm that takes sequence data as input and recursively in the direction of sequence evolution, its nodes are connected in a chain, and it is featured with memory, parameter sharing, and Turing completeness [17, 18]. It has great advantages when learning the nonlinear features of the sequence. Under its optimization, ultrasound elastography can monitor the effect of disease treatment more accurately [19]. In this study, 108 patients with cervical cancer were selected and randomly divided into an experimental group with 55 patients treated with CRISPR and shRNA plasmid nanoparticle drugs and a control groups (53 patients) treated with the same amount of normal saline. The ultrasound elastography image results before and after the administration of the two groups were collected and compared to analyze the effect of CRISPR shRNA nanoparticles in the treatment of cervical cancer, aiming to provide data support and theoretical guidance for the clinical diagnosis and treatment of cervical cancer. The results showed that the experimental group had a total of 217 points before drug administration and a total of 224 points after drug administration. Each patient had an average increase of 0.13 points. The control group had a total of 200 points before drug administration and a total of 223 points after drug administration. Each patient had an average increase of 0.43 points. Therefore, the experimental group was significantly different from the control group (*P* < 0.05). Each organization chart output by the RNN algorithm was clearer than the original image, and the score given by intelligent calculation was faster than that of professional doctors. The monitoring effect of the deep learning RNN intelligent algorithm on the therapeutic effect of nanomedicine was analyzed, it was found that the average accuracy of the experimental group and control group was 98.95% and 90.34%, respectively, and the experimental group was significantly different from the control group (*P* < 0.05). Such results were consistent with the research conclusions of Zhu et al. [20]. Nano-CRISPR and shRNA drugs are effective in the treatment of cervical cancer. Nano-CRISPR and shRNA drugs have significant effects on the treatment of cervical cancer, and the scores given by the deep learning intelligent algorithm are faster and more accurate, which can provide theoretical guidance for the clinical application of deep learning algorithms to analyze the treatment effects of diseases.

## 5. Conclusion

In this study, CRISPR and shRNA plasmid nanoparticle drugs were used to treat patients with cervical cancer, and the effects of nanoparticle treatment on cervical cancer were compared with those without drug treatment. In addition, the scores for cervical cancer were given by doctors and RNN intelligent algorithms to compare and analyze the monitoring effect of deep learning on the therapeutic effect of nanomedicine. The results suggested that nano-CRISPR and shRNA drugs can effectively inhibit the growth of cervical cancer cells and had a significant effect on the treatment of cervical cancer; each tissue image output by the deep learning algorithm was clearer and more identifiable, and the score given by the intelligent algorithm calculation was faster and more accurate. However, the sample size selected in this study was relatively small, which may have a certain impact on the final results of the experiment. Therefore, in future experiments, it is necessary to expand the sample size and further analyze and compare the effects of ultrasound elastography of deep learning algorithms on the treatment of cervical cancer with CRISPR shRNA nanoparticles. In short, it provided theoretical guidance for the clinical application of deep learning to analyze the therapeutic effect of diseases.

## Data Availability

The data used to support the findings of this study are available from the corresponding author upon request.
